# Investigation of Healthcare Professionals’ Knowledge of Evidence-Based Clinical Practices for Preterm Neonatal Skin Care—A Pilot Study

**DOI:** 10.3390/children9081235

**Published:** 2022-08-16

**Authors:** Dimitra Metallinou, Christina Nanou, Panagiota Tsafonia, Grigorios Karampas, Katerina Lykeridou

**Affiliations:** 1 Department of Midwifery, University of West Attica, 12243 Athens, Greece; 2 Obstetrics and Gynaecology Clinic, General Hospital of Agrinio, 30133 Agrinio, Greece; 3 Second Department of Obstetrics and Gynaecology, Aretaieio Hospital, National and Kapodistrian University of Athens, 11528 Athens, Greece

**Keywords:** healthcare professionals, theoretical knowledge, clinical knowledge, evidence-based, clinical practices, preterm, neonatal skin care

## Abstract

Neonatal skin care practices are considered crucial for a neonate’s survival and are closely related to healthcare professionals’ (HPs) knowledge and skills in delivering scientifically valid neonatal care interventions. In this descriptive cross-sectional pilot study, conducted in 2022, we aimed to assess HPs’ basic theoretical knowledge of neonatal vernix caseosa, skin microbiota, and bathing as well as knowledge regarding evidence-based clinical practices (referred to as “clinical knowledge”) for preterm neonatal skin care. Eligible participants were neonatologists, pediatricians, obstetricians, midwives and nurses working in the Greek setting. The research instrument was an online questionnaire designed by the research team. Finally, 123 HPs took part in the study. The theoretical, clinical and total knowledge scores were all significantly associated with age, healthcare profession and the sources used for education. Participants’ theoretical and clinical knowledge scores were compared and found not to differ significantly (*p* = 0.566). A significant and positive correlation was found between theoretical and clinical knowledge scores. Thus, it is concluded that HPs should be updated with the latest evidence-based knowledge and clinical guidelines in order to provide neonatal skin care with high-quality standards.

## 1. Introduction

The skin is the body’s largest organ with quite an essential role during the neonatal period as it regulates fluid balance, maintains thermoregulation and protects the neonate from infection. More recent evidence supports the skin barrier continuously developing within the first two years of life, and thus optimal skin care is critical from the early neonatal period to toddlerhood [1].

For those neonates born preterm, the skin can be quite immature, especially in the first 2 weeks of life and therefore susceptible to damage. As a consequence, there is an increased risk for invasion by microorganisms and thus morbidity and mortality [2]. During hospitalization, maintenance of skin integrity, the decrease of risk factors and neonatal skin care education for parents/caregivers are key priorities for the multidisciplinary team that takes care of the preterm neonate [3]. That team, mainly consisting of healthcare professionals (HPs) such as pediatricians/neonatologists, midwives and nurses, should be updated with the latest evidence-based knowledge to provide neonatal skin care with high-quality standards. Such knowledge should include assessment of the neonate’s skin condition, identification of high-risk neonates for disruption of normal skin barrier function, recognition of environmental factors and treatment-related agents that may affect neonatal skin integrity as well as interventions to promote optimal skin function and reduce skin trauma [4]. Assurance of high-quality services is provided by the implementation of revised clinical practice guidelines based on the best available evidence.

Previous studies have evaluated knowledge level, experience, practices and beliefs of the nurses working in neonatal units regarding preterm neonatal skin health. Interestingly, the participants in these studies expressed concern about the inadequacy of available evidence and the limited awareness of the subject [5,6]. More specifically, Liversedge et al. [5] reported discrepancies in the participants’ responses, a fact that indicates insufficiency in education and evidence-based clinical practices, while Mohamed et al. [6] demonstrated gaps in participants’ theoretical and practical knowledge of preterm neonatal skin care. In contrast to these studies, we decided to recruit HPs with different employment statuses and working positions in different levels of healthcare instead of neonatal unit staff to investigate knowledge of evidence-based clinical practices for preterm neonatal skin care. We did this because in Greece, a country that has adopted a highly centralized “mixed health system” model [7], the private sector is expanded and plays an important role in the provision of health services, especially in maternal and neonatal care. This means that mothers usually collaborate with private HPs who continuously provide counseling and support during the perinatal period. In the occurrence of an unexpected event, such as a preterm birth, Greek women and their family often consult as a coping mechanism these already known healthcare providers for the care of the preterm neonate. The neonatal unit staff is commonly asked subsequently for advice, as it might take a little more time for them to gain parental confidence, overcome communication gaps, build/maintain relationships, exchange information and share decision-making with parents [8]. Similar behaviors are also observed in women who attend public perinatal care services, as circumstantial evidence implies that informal payments are still common in Greek public hospitals and women usually cooperate with and refer to specific HPs [7]. As a result, we are of the opinion that all perinatal HPs should be updated and in line with the latest guidelines because appropriate neonatal skin care practices are considered crucial for a neonate’s survival and are closely related to HP’s knowledge and skills to deliver scientifically valid neonatal care interventions.

Taking into consideration the Greek healthcare framework and typical Greek behavioral aspects, we aimed to assess HPs’ (a) basic theoretical knowledge of neonatal vernix caseosa, skin microbiota and bathing and (b) knowledge regarding evidence-based clinical practices for preterm neonatal skin care (referred to as “clinical knowledge” for the needs of the present study). We hypothesized that significant and positive correlations between theoretical and clinical knowledge would be found.

## 2. Materials and Methods

### 2.1. Study Design and Participants

This is a descriptive cross-sectional study conducted in June 2022. Ethical approval for this study was obtained from the Ethics Committee of the University of West Attica (IRB R.No.: 53752/08-06-2022), which is located in Attica, the capital of Greece.

Eligible participants were HPs who worked with neonates, either term or preterm, including neonatologists, pediatricians, obstetricians, midwives and nurses. The HPs needed to speak and understand the Greek language fluently in order to be able to complete the questionnaire of the study. During the recruitment period, numerous HPs all over Greece were invited via email and social media to join the study.

### 2.2. Procedure of Recruitment

An invitation letter with a link to the survey was posted by the research team on social media platforms for professionals or emailed to the perinatal HPs of Greece, members of professional networks or employees at hospitals/clinics. In the letter, the nature and the purpose of the study was explained, helping to create interest and eagerness to serve as research subjects. Once the participant voluntarily agreed and consented to participate online, the questionnaire was unlocked. Recruitment was in compliance with all ethical principles and participation in the study entailed no direct personal benefits.

The questionnaire was administered online mainly due to COVID-19 restrictions, time constraints and with a view to prevent systematic errors as a result of postal handling procedures. A code was assigned automatically to each participant by the database used so that the de-identification could be preserved.

### 2.3. Research Instrument

The research instrument was one questionnaire divided into three sections. The first section referred to the demographic and occupational characteristics of gender, age, educational/employment status, healthcare profession, total working experience, geographical area and level of healthcare of current work. The second section consisted of 21 questions that explored theoretical knowledge about neonatal vernix caseosa, skin microbiota and bathing. The third section consisted of 21 questions that assessed the knowledge of HPs regarding evidence-based clinical practices for preterm neonatal skin care. Both theoretical and clinical knowledge were the knowledge derived from the latest available evidence-based, published literature.

The questions for all sections were formulated by the research team following a comprehensive review of the relevant literature. The questionnaire was generated from well-respected sources reporting evidenced-based guidelines or recommending evidence-based practices for optimal neonatal skin care [1,9,10,11,12,13]. Questions were then piloted, redefined and validated by a group of experts that consisted of two neonatologists and two midwives with working experience in a neonatal intensive care unit (NICU) of more than 15 years. The final form of the questionnaire was approved by the research team.

All questions included in the questionnaire were close-ended and were either true/false questions or single-answer multiple choice. Each question needed to be answered within 60 s.

### 2.4. Statistical Analysis

Quantitative variables were expressed as mean values (standard deviation), while qualitative variables were expressed as absolute and relative frequencies. Student’s *t* tests and analysis of variance (ANOVA) were computed for the comparison of mean values. Bonferroni correction was used in order to control for type I errors. Pearson correlation coefficients were used to investigate the association of two continuous variables. Paired Student’s *t* test was used for the comparison between theoretical and clinical knowledge. Multivariate linear regression analyses were conducted in order to identify demographic and occupational characteristics that were independently associated with participants’ knowledge scores. All reported *p* values are two-tailed. Statistical significance was set at *p* < 0.05 and analyses were conducted using SPSS statistical software (version 22.0, IBM, Armonk, NY, USA).

## 3. Results

### 3.1. Participants’ Demographic and Occupational Characteristics

The final study sample consisted of 123 HPs. Participants’ demographic and occupational characteristics are presented extensively in Table 1. Regarding the HPs’ theoretical knowledge of neonatal vernix caseosa, skin microbiota and bathing, as well as of clinical practices for neonatal skin care, this was mainly derived from professional experience (52.0%) in comparison to other sources (Table 1).

### 3.2. Participants’ Theoretical Knowledge about Neonatal Vernix Caseosa, Skin Microbiota and Bathing

The questions that assessed HPs’ theoretical knowledge about neonatal vernix caseosa, skin microbiota and bathing, along with their performance, are broadly presented in the table below (Table 2).

The percentages for correct answers ranged from 18.7 to 97.6%. More specifically, few participants answered correctly that ‘the microbial flora of the neonatal skin is more diverse in dry skin areas’ and that ‘neonatal skin microbiota is initially established 3–4 days after birth’. Additionally, the majority of respondents correctly answered that ‘the NICU environment can affect the development of skin microbiota in preterm neonates and that ‘intravenous antibiotics can affect neonatal skin microbiota’.

Participants’ correct answers were then added. This sum was converted into a 0–100 scale. Thus, the theoretical knowledge score could range from 0% to 100%, with higher scores indicating greater knowledge. Total scores varied from 28.57% to 100% with the mean value being 59.47% (SD = 14.8%). None of the participants scored zero (i.e., did not answer any questions correctly) and 1 person (0.8%) scored 100 (i.e., answered all questions correctly).

Furthermore, participants’ theoretical knowledge score was associated with their demographic and occupational characteristics. Results are depicted in Table 3.

Participants’ theoretical knowledge score was significantly associated with age, healthcare profession, geographical area of current work and the sources used for acquiring knowledge about neonatal vernix caseosa, skin microbiota, bathing and clinical practices for neonatal skin care. More specifically, after the Bonferroni correction it was found that participants aged 20–40 years old and 41–50 years old had significantly lower scores, indicating lower knowledge of the theme compared to those aged 51 and above (*p* = 0.003 and *p* = 0.043 respectively). Further analysis showed that participants who had gained the aforementioned knowledge from their undergraduate or postgraduate studies had significantly lower scores in comparison with participants who focused on personal study and research (*p* = 0.010). Not surprisingly, significantly higher scores, indicating greater knowledge, were observed among doctors and, interestingly, among participants working outside the prefecture of Attica.

### 3.3. Participants’ Knowledge Regarding Evidence-Based Clinical Practices for Preterm Neonatal Skin Care

Information about participants’ knowledge regarding evidence-based clinical practices for preterm neonatal skin care are presented in the following table (Table 4).

The percentages for correct answers ranged from 14.6 to 98.4%. More specifically, a minority of participants answered correctly that ‘to reduce stress in preterm neonates the recommended bathing technique is swaddled immersion bath’ and that ‘in preterm neonates, in order to protect the stratum corneum, the nappy area should be cleaned only with water for the first 4 weeks of life’. Additionally, when participants were asked about ‘the use of inappropriate detergents in the linen and clothing’, most of them answered correctly that in preterm neonates ‘may affect neonatal skin microbiome’. At last, 95.1% of the subjects commented accurately that ‘in preterm neonates, application of protective dressings is suggested when nasal CPAP devices are used’.

Participants’ correct answers were added subsequently. This sum was converted into a 0–100 scale. Total scores ranged from 28.57 to 100% with the mean value being 60.12% (SD = 13.34%). None of the participants scored zero and 2 participants (1.6%) scored 100.

Participants’ clinical knowledge score was then associated with demographic and occupational characteristics (Table 5).

Participants’ clinical knowledge score was significantly associated with age, healthcare profession and the sources used for acquiring knowledge on neonatal vernix caseosa, skin microbiota, bathing and clinical practices for neonatal skin care. More specifically, after the Bonferroni correction it was found that participants aged 20–40 years old had significantly lower scores compared to those who were at least 51 years old (*p* = 0.015). Moreover, it was observed that participants who learned about neonatal vernix caseosa, skin microbiota, bathing and clinical practices for neonatal skin care from personal study and research had significantly higher scores compared to those who gained knowledge from undergraduate or postgraduate studies (*p* < 0.001), professional experience (*p* = 0.006) or seminars/congresses/lectures/courses (*p* = 0.003). As could be expected, doctors demonstrated significantly higher scores, indicating greater knowledge of the topic.

### 3.4. Total Knowledge Score

All theoretical and clinical correct answers were added for each participant. The total knowledge score of the participants was found to be between 30.95% and 97.62% with the mean value being 59.79% (SD = 12.63%). None of the participants scored zero or 100. Associations among participants’ total knowledge score and demographic and occupational characteristics are presented in Table 6.

Participants’ total knowledge score was significantly associated with age, healthcare profession and sources used for acquiring knowledge on neonatal vernix caseosa, skin microbiota, bathing and clinical practices for neonatal skin care. More specifically, after the Bonferroni correction, it was found that participants aged 20–40 years old and 41–50 years old had significantly lower scores compared to those who were at least 51 years old (*p* = 0.002 and *p* = 0.024 respectively). In addition, it was found that participants who learned about neonatal vernix caseosa, skin microbiota, bathing and clinical practices for neonatal skin care from personal study and research had significantly higher scores compared to those who had learned about the topic from their undergraduate or postgraduate studies, professional experience and seminars/congresses/lectures/courses or other sources (*p* < 0.001, *p* = 0.009 and *p* = 0.007 respectively). At last, doctors demonstrated significantly higher scores, indicating greater knowledge.

### 3.5. Demographic and Occupational Characteristics Independently Associated with Participants’ Theoretical, Clinical and Total Knowledge Scores

Multivariate linear regression analysis was conducted in order to identify demographic and occupational characteristics independently associated with participants’ theoretical, clinical and total knowledge scores (Tables S1–S3).

Participants’ age, healthcare profession and sources used for acquiring knowledge on vernix caseosa, skin microbiota, neonatal bathing and clinical practices for neonatal skin care were found to be independently associated with their clinical and total knowledge score, while their theoretical knowledge score was independently associated only with age and healthcare profession.

In particular, participants who were at least 51 years old when compared to participants aged 20–40 years old were found to demonstrate significantly higher scores in theoretical, clinical and total knowledge by 10.85, 11.12 and 10.99 units, respectively. Moreover, doctors when compared to midwives and nurses were found to have significantly higher scores in theoretical, clinical and total knowledge by 11.85, 11.42 and 11.64 units, respectively. Finally, participants who had learned about neonatal vernix caseosa, skin microbiota, bathing and clinical practices for neonatal skin care from personal study and research had significantly higher scores in clinical and total knowledge by 11.74 and 9.88 units, respectively, compared to those who had learned about the topic from their undergraduate or postgraduate studies.

### 3.6. Comparison between Theoretical and Clinical Knowledge Scores

**Figure 1:**
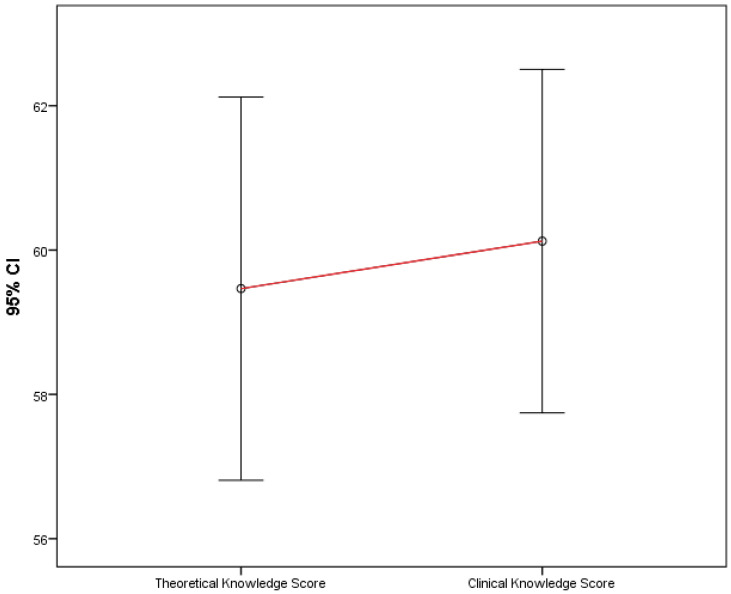
Comparison of theoretical and clinical knowledge scores.

Participants’ theoretical and clinical knowledge scores were compared and found not to differ significantly (*p* = 0.566), as shown in Figure 1.

### 3.7. Correlation between Theoretical and Clinical Knowledge Scores

Pearson’s correlation coefficient (r) between theoretical and clinical knowledge scores is presented in Table 7.

**Figure 2:**
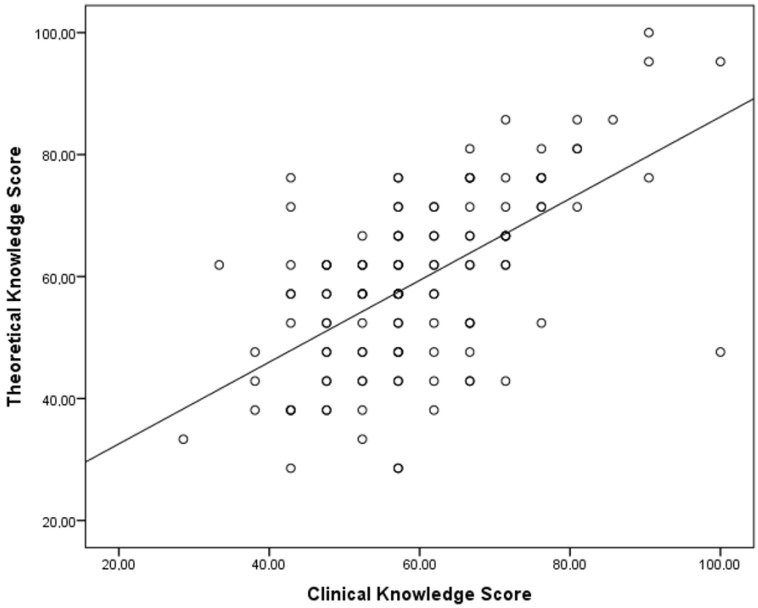
Correlation between theoretical and clinical knowledge scores.

A significant and positive correlation was found between theoretical and clinical knowledge scores. Thus, greater theoretical knowledge was significantly associated with greater clinical knowledge (Figure 2).

## 4. Discussion

The present study, as far as we know, is the first to identify HPs’ knowledge of evidence-based clinical practices for preterm neonatal skin care in the Greek setting. Previous research from the UK [5] demonstrated the beliefs and practices of nurses working in neonatal units in which participants stated that formal skin care training was severely lacking, and instead, bedside training was offered from the more experienced colleagues to the junior staff. This finding is in agreement with our own observation, where 52% of the participants reported that basic theoretical knowledge about neonatal skin and its care is mainly derived from professional experience in comparison to other sources of education. However, the present study was conducted in the midst of the COVID-19 pandemic, so it cannot be ruled out that it had a strong impact on teaching and learning in health professional education [14], including the non-occurrence of educational courses. The development and presence of continuing educational programs in neonatal units and operational frameworks for primary healthcare maintain current practice, translate knowledge into clinical implementation, connect newly acquired knowledge and skills to what is already known or experienced and thus sustain an HP’s career [15,16].

In our study, participants’ total scores in theoretical knowledge about neonatal vernix caseosa, skin microbiota and bathing varied from 28.57% to 100%. Such an observation can be explained by the main differences in the study sample (age, specialty, sources of education). Nevertheless, the highest scores indicating greater knowledge were observed in questions relating to NICU environment and intravenous antibiotics use, showing that HPs were aware of key factors that affect mainly preterm neonates and neonatal skin microbiota. Primary caregivers in a NICU setting and during the neonatal period should be updated to the best available research to apply clinical practices that protect neonatal skin microbiome as its development is critical, having long-term impacts [17].

As far as HPs’ clinical and total knowledge are concerned, these were significantly associated with age and profession specialty but also with sources of education. Participants who educated themselves on the topic by personal study and research had significantly higher scores compared to other sources of education. This finding highlights the fact that for HPs it is very important to develop independence in determining their learning needs as well as to ascertain when and how these needs can be addressed. Healthcare professionals are continuous learners, and self-study skills are distinguishing traits of practitioners who want to be professionally competent [18].

Furthermore, participants’ theoretical and clinical knowledge scores were found not to differ significantly and a positive correlation between theoretical and clinical knowledge scores was confirmed. These results demonstrate the absence of a knowledge gap between theoretical and clinical knowledge concerning neonatal skin care, especially the preterm, possibly due to the fact that a large percentage had acquired an MSc or PhD degree (67.5%) and 36.6% had more than 10 years of experience. Several studies, though [6,19,20], have found barriers that utilize theoretical knowledge in clinical settings, such as disbelief in clinical competence, deficiencies in the teaching and learning process and differences between simulation-based learning and real-life clinical situations.

This pilot study has gone some way towards enhancing our understanding of the HPs’ knowledge of evidence-based clinical practices for preterm neonatal skin care, especially in the Greek setting. We are aware, though, that our research had some strengths and limitations. A major strength was that participants came from health facilities of different levels of care, which belonged to either the public or the private sector, and were located both in the capital and the provinces, indicating a more representative sample in terms of knowledge. We acknowledge, though, that these are preliminary findings of a pilot study and as a consequence their generalization in the whole Greek context is restricted. Another strength of our study was that our questionnaire was based on the latest available evidence-based research [1,9,10,11,12,13] and exploring whether HPs are in line with it. Furthermore, because the present study was conducted online via email and social media invitation, a main limitation is that the response rate cannot be calculated. Moreover, the questionnaire’s section which assessed HPs’ theoretical knowledge of ‘neonatal vernix caseosa, skin microbiota, bathing and clinical practices for neonatal skin care’, included questions both for term and preterm neonates, creating a difficulty in strictly investigating knowledge about preterm neonatal skin care. Lastly, participants in our study had different specialties and it was not mandatory to belong to neonatal units’ staff, making our results difficult to compare with other studies which recruited only experienced neonatal staff.

The lessons learned from this pilot study will undoubtedly improve the research design of further inquiries. Future researchers should consider expanding resources and develop enough evidence for subsequent studies. We recommend that further research should recruit a larger number of HPs so as to achieve a representative sample. A flexible recruitment plan which allows modifications when unexpected barriers appear should be developed as well. Procedures of data collection should be well defined to demonstrate validity and reliability [21].

The feasibility of assessing HPs’ knowledge, in relation to preterm neonatal skin care, according to the level of healthcare facility they work or their specialty/subspecialty, should be examined, also taking into account how much time during the day the HP works with preterm neonates. Additionally, the measurement tool was relatively new, so this pilot study provided direction for its refinement [22], mainly regarding the second section which investigated theoretical knowledge. It is proposed that future work should focus on NICU staff as preterm neonates are more likely to require prolonged hospitalization and therefore be in need of supportive care that may increase the risk of skin injury. By gaining a wider perspective on whether knowledge gaps in preterm infants’ skin care exist among NICU staff, it will bring to light the necessity or not of developing clinical evidence based guidelines on preterm infant skin care in a national level so as to promote consistency in practice within the contexts of NICUs.

Finally, future studies need to investigate the applicability of reported evidence-based research to the clinical setting and their potential effect on care outcomes, by ensuring that guidelines are efficiently disseminated and implemented [23]. This will provide direction for incorporating educational intervention and continuing professional support with the goal to optimize preterm neonatal skin care.

## 5. Conclusions

Our work has led us to conclude that knowledge of evidence-based clinical practices for preterm neonatal skin care is an under-reported problem. Further research is needed to evaluate if HPs work along with evidence-based guidelines in their daily clinical practice. Implementing clinical practice guidelines demands a systematic planning process with a focus on institutional and social context, barriers and facilitators that arise from the introduction of innovations or changes of proven value in specific environments and finally, assessment of intervention strategies for the implementation to be efficient, effective, safe and patient-centered.

## Figures and Tables

**Table 1:** Participants’ demographic and occupational characteristics.

	*N*	%
Gender		
Male	30	24.4
Female	93	75.6
Age		
20–30	16	13.0
31–40	34	27.6
41–50	44	35.8
51–60	24	19.5
61 and above	5	4.1
Healthcare profession		
Midwife	65	52.8
Nurse	1	0.8
Obstetrician	24	19.5
Pediatrician	25	20.3
Neonatologist	8	6.5
Educational status		
University degree	0	0.0
Technical university degree	40	32.5
MSc	45	36.6
PhD	38	30.9
Employment status		
Employee in private sector	23	18.7
Employee in public sector	83	67.5
Self-employed	17	13.8
Academic	0	0.0
Total working experience		
0–5 years	28	22.8
6–10 years	22	17.9
11–15 years	22	17.9
16–20 years	23	18.7
More than 20 years	28	22.8
Geographical area of current work		
Within the prefecture of Attica	22	17.9
Outside the prefecture of Attica	101	82.1
Level of healthcare of current work		
Primary healthcare	33	26.8
Secondary healthcare	55	44.7
Tertiary healthcare	35	28.5
Your theoretical knowledge about neonatal vernix caseosa, skin microbiota, bathing and clinical practices for neonatal skin care mainly derives from		
Undergraduate studies	21	17.1
Postgraduate studies	2	1.6
Professional experience	64	52.0
Personal study and research	19	15.4
Seminars/Congresses/Lectures/Courses	13	10.6
Other source	4	3.3

**Table 2:** Questions and healthcare professionals’ performance regarding theoretical knowledge about neonatal vernix caseosa, skin microbiota and bathing.

	*N*	%	Correct Answer (%)
Vernix Caseosa is Composed of			49.6
water (90%)–lipids (5%)–proteins (5%)	8	6.5	
water (80%)–lipids (10%)–proteins (10%) *	61	49.6	
water (70%)–lipids (20%)–proteins (10%)	37	30.1	
water (60%)–lipids (20%)–proteins (20%)	17	13.8	
There are no differences in the lipid composition of vernix caseosa and the stratum corneum.			71.5
True	35	28.5	
False *	88	71.5	
Vernix caseosa is			78.0
Hydrophobic *	96	78.0	
Hydrophilic	27	22.0	
Vernix caseosa is more vapor-permeable than stratum corneum			40.7
True	73	59.3	
False *	50	40.7	
The preservation of the vernix caseosa facilitates formation of the acid mantle of the neonatal skin.			91.1
Right *	112	91.1	
Wrong	11	8.9	
Vernix caseosa has			87.0
Antimicrobial and healing properties	9	7.3	
Thermoregulation properties	7	5.7	
Antioxidant properties	0	0.0	
All the above *	107	87.0	
None of the above	0	0.0	
The anti-inflammatory properties of the vernix caseosa are related to			30.9
Fungi	1	0.8	
Bacteria	5	4.1	
Viruses	0	0.0	
Fungi and bacteria *	38	30.9	
All the above	79	64.2	
The stratum corneum (the outer layer of the skin) of term neonates consists of			36.0
2–4 layers	14	11.6	
4–8 layers	36	29.5	
10–20 layers *	44	36.0	
20–30 layers	28	23.0	
The stratum corneum (the outer layer of the skin) of an extremely preterm neonate consists of			52.0
2–3 layers *	64	52.0	
7–8 layers	49	39.8	
10–12 layers	5	4.1	
13–15 layers	5	4.1	
Neonatal skin maturation continues until the age of			53.7
30 days of life	17	13.8	
3 months	15	12.2	
6 months	25	20.3	
12 months *	66	53.7	
The use of incubator humidity during the first 2 weeks of life in extremely preterm neonates			85.8
Increases transepidermal water loss	17	14.2	
Decreases transepidermal water loss *	105	85.8	
Transepidermal water loss in preterm neonates is increased due to			66.3
Insufficiency of the stratum corneum *	81	66.3	
Insufficient subcutaneous fat stores	41	33.7	
Neonatal skin microbiota is initially established			23.6
Immediately after birth	80	65.0	
3–4 days after birth *	29	23.6	
1 month after birth	13	10.6	
1 year after birth	1	0.8	
Initial colonization of the neonatal skin is dependent on			86.2
Mode of delivery *	106	86.2	
Feeding type	6	4.9	
Skin care	11	8.9	
Early–life neonatal skin microbiota varies significantly among different body sites			26.8
True	90	73.2	
False *	33	26.8	
The microbial flora of the neonatal skin is more diverse in			18.7
Moist skin areas	34	27.6	
Dry skin areas *	23	18.7	
Sebacous skin areas	66	53.7	
Intravenous antibiotics can affect neonatal skin microbiota			93.9
True *	115	93.9	
False	7	6.1	
The NICU environment can affect the development of skin microbiota in preterm neonates			97.6
True *	120	97.6	
False	3	2.4	
Dysbiosis of neonatal skin microbiota can cause			72.4
Eczema/atopic dermatitis	27	22.0	
Seborrheic dermatitis	3	2.4	
Asthma	0	0.0	
All the above *	89	72.4	
None of the above	4	3.3	
The corneal blink reflex, which importantly protects the neonatal eye during bathing, matures fully after			45.5
7 days of life	15	12.2	
14 days of life	11	8.9	
30 days of life	41	33.3	
120 days of life *	56	45.5	
The first bathing of a neonate whose mother is HIV positive must occur			43.1
Immediately after birth	50	40.7	
As soon as possible after birth *	53	43.1	
6–24 h after birth	20	16.3	

* indicates correct answer.

**Table 3:** Associations among participants’ theoretical knowledge score and demographic and occupational characteristics.

		Theoretical Knowledge Score		
		Mean	SD	*p* ANOVA
Gender	Male	59.52	16.10	0.981 *
	Female	59.45	14.55	
Age	20–40	55.71	11.79	0.003
	41–50	58.66	16.10	
	51 and above	67.16	15.34	
Healthcare profession	Midwife/Nurse	56.06	15.69	0.006 *
	Obstetrician/Pediatrician/Neonatologist	63.41	12.92	
Educational status	Technical university degree	57.02	15.90	0.443
	Master’s degree	60.32	11.31	
	Doctoral degree	61.03	17.36	
Employment status	Employee in private sector	55.28	14.69	0.327
	Employee in public sector	60.36	14.61	
	Self-employed	60.78	16.28	
Total working experience	0–5 years	59.18	11.11	0.223
	6–10 years	54.11	13.91	
	11–15 years	57.58	17.20	
	16–20 years	62.53	15.94	
	More than 20 years	62.93	15.57	
Geographical area of current work	Within the prefecture of Attica	53.90	14.50	0.050 *
	Outside the prefecture of Attica	60.68	14.75	
Level of healthcare of current work	Primary healthcare	58.44	15.53	0.106
	Secondary healthcare	62.42	14.42	
	Tertiary healthcare	55.78	14.41	
Your theoretical knowledge about neonatal vernix caseosa, skin microbiota, bathing and clinical practices for neonatal skin care mainly derives from	Undergraduate / Postgraduate studies	54.04	13.67	0.013
	Professional experience	59.45	14.07	
	Personal study and research	68.42	18.04	
	Seminars/Congresses/Lectures/ Courses/Other source	56.86	11.84	

* *p*-value from independent samples *t* test.

**Table 4:** Questions and healthcare professionals’ performance regarding evidence-based clinical practices for preterm neonatal skin care.

	N	%	Correct Answer (%)
The skin surface pH of preterm neonates in the first day of life is			65.9
>6 *	81	65.9	
6	20	16.3	
<6	22	17.9	
The skin surface pH of preterm neonates reaches the value of 5			28.5
At the end of the first week of life	30	24.4	
At the end of the second week of life	58	47.2	
At the end of the third week of life *	35	28.5	
What percentage does the skin itself provide of the body weight of a preterm neonate?			64.2
3	26	21.1	
13 *	79	64.2	
23	18	14.6	
Neonates with gestational age < 32 weeks once cardioraspiratory and thermal stability is achieved, can be bathed for the first time after			45.5
1st–2nd day of life	8	6.5	
3rd–5th day of life *	56	45.5	
5th–7th day of life	44	35.8	
9th–10th day of life	15	12.2	
In neonates with gestational age < 32 weeks the use of appropriate cleansers is safe after			65.0
24 h of life	7	5.7	
2–3 days of life	16	13.0	
The first week of life *	80	65.0	
The first 10 days of life	20	16.3	
To reduce stress in preterm neonates the recommended bathing technique is			14.6
Warm wet washcloths inside the incubator	68	55.3	
Immersion bath	37	30.1	
Swaddled immersion bath *	18	14.6	
In preterm neonates.in order to protect the stratum corneum, the nappy area should be cleaned only with water for the first			25.2
10 days of life	35	28.5	
2 weeks of life	51	41.5	
4 weeks of life *	31	25.2	
8 weeks of life	6	4.9	
In preterm neonates a barrier cream for the nappy area should be applied			44.7
At each nappy change	44	35.8	
Since the first signs of redness appear *	55	44.7	
In severe nappy rash	24	19.5	
In preterm neonates, red, scaly skin with excoriations in the groin and neck folds may be due to lack of			42.3
Iron	6	4.9	
Zinc *	52	42.3	
Vitamin E	65	52.8	
To maintain the skin integrity of preterm neonates it is indicated the use of emollient with			30.1
Sunflower oil *	37	30.1	
Cedar oil	7	5.7	
All the above	20	16.3	
None of the above	59	48.0	
In preterm neonates, the use of inappropriate detergents in the linen and clothing may affect neonatal skin microbiome			98.4
Right *	121	98.4	
Wrong	2	1.6	
Prophylactic emollient use in preterm neonates weighing 750 gr or less is associated with an increased risk of infection			91.1
True *	112	91.1	
False	11	8.9	
In extremely preterm neonates the epidermal barrier function during the first days of life can be promoted through			67.5
The use of appropriate moisturizing products	2	1.6	
Skin to skin contact with the parent	38	30.9	
The use of appropriate incubator humidification *	83	67.5	
In preterm neonates, when there is an open wound on the skin, this should be			73.2
Covered with sterile material in order to prevent infection *	90	73.2	
Left uncovered to “air-out” and thus heal faster	33	26.8	
In preterm neonates with gestational age < 32 weeks, the use of inotropes is a risk factor for skin breakdown			84.6
True *	104	84.6	
False	19	15.4	
Monitor probes can lead to local skin necrosis due to pressure in preterm neonates with oedematous dermis			90.2
True *	111	90.2	
False	12	9.8	
In preterm neonates, the use of skin-protective film is suggested before the use of adhesive dressings and tapes			87.8
True *	108	87.8	
False	15	12.2	
In preterm neonates, adhesive tapes are suggested to be removed with			92.7
Warm water only *	114	92.7	
Antiseptic solution	5	4.1	
Alcoholic solution	4	3.3	
In preterm neonates, intravenous lines should be observed for signs of infiltration every			26.0
1 h *	32	26.0	
3 h	72	58.5	
6 h	10	8.1	
8 h	9	7.3	
In preterm neonates, application of protective dressings is suggested when nasal CPAP devices are used			95.1
True *	117	95.1	
False	6	4.9	
Neonatal Skin Risk Assessment Scales in preterm neonates admitted to the NICU should be completed			30.1
Upon admission	77	62.6	
Within 2 h of admission *	37	30.1	
Within 12 h of admission	3	2.4	
Within 24 h of admission	6	4.9	

* indicates correct answer.

**Table 5:** Associations among participants’ clinical knowledge score and demographic and occupational characteristics.

		Clinical Knowledge Score		
		Mean	SD	*p* ANOVA
Gender	Male	60.00	12.54	0.954 *
	Female	60.16	13.65	
Age	20–40	57.52	10.13	0.016
	41–50	59.09	12.72	
	51 and above	66.17	17.19	
Healthcare profession	Midwife/Nurse	57.43	12.94	0.015 *
	Obstetrician/Pediatrician/Neonatologist	63.24	13.22	
Educational status	Technical university degree	58.10	12.63	0.079
	Master’s degree	58.52	10.23	
	Doctoral degree	64.16	16.41	
Employment status	Employee in private sector	58.80	14.47	0.494
	Employee in public sector	59.78	13.94	
	Self-employed	63.59	7.52	
Total working experience	0–5 years	58.67	10.92	0.351
	6–10 years	59.31	9.49	
	11–15 years	57.36	13.59	
	16–20 years	65.01	16.16	
	More than 20 years	60.37	15.12	
Geographical area of current work	Within the prefecture of Attica	58.44	15.14	0.516 *
	Outside the prefecture of Attica	60.49	12.96	
Level of healthcare of current work	Primary healthcare	59.60	12.77	0.946
	Secondary healthcare	60.09	13.36	
	Tertiary healthcare	60.68	14.16	
Your knowledge about neonatal vernix caseosa, skin microbiota, bathing and clinical practices for neonatal skin care mainly derives from	Undergraduate/Postgraduate studies	55.07	12.08	<0.001
	Professional experience	59.82	11.81	
	Personal study and research	70.93	16.56	
	Seminars/Congresses/Lectures/ Courses/Other source	56.02	10.18	

* *p*-value from independent samples *t* test.

**Table 6:** Associations among participants’ total knowledge score and demographic and occupational characteristics.

		Total Knowledge Score		
		Mean	SD	*p* ANOVA
Gender	Male	59.76	13.21	0.987 *
	Female	59.81	12.50	
Age	20–40	56.62	9.00	0.002
	41–50	58.87	13.36	
	51 and above	66.67	14.50	
Healthcare profession	Midwife/Nurse	56.75	12.71	0.004 *
	Obstetrician/Pediatrician/Neonatologist	63.32	11.67	
Educational status	Technical university degree	57.56	12.86	0.207
	Master’s degree	59.42	9.66	
	Doctoral degree	62.59	15.06	
Employment status	Employee in private sector	57.04	13.32	0.422
	Employee in public sector	60.07	12.91	
	Self-employed	62.18	9.96	
Total working experience	0–5 years	58.93	9.25	0.282
	6–10 years	56.71	9.90	
	11–15 years	57.47	14.66	
	16–20 years	63.77	13.94	
	More than 20 years	61.65	14.25	
Geographical area of current work	Within the prefecture of Attica	56.17	12.85	0.138
	Outside the prefecture of Attica	60.58	12.50	
Level of healthcare of current work	Primary healthcare	59.02	12.98	0.501
	Secondary healthcare	61.26	12.89	
	Tertiary healthcare	58.23	11.95	
Your knowledge about neonatal vernix caseosa, skin microbiota, bathing and clinical practices for neonatal skin care mainly derives from	Undergraduate / Postgraduate studies	54.55	10.89	0.001
	Professional experience	59.64	11.36	
	Personal study and research	69.67	15.85	
	Seminars/Congresses/Lectures/ Courses/Other source	56.44	9.70	

* *p*-value from independent samples t-test.

**Table 7:** Correlation between theoretical and clinical knowledge scores.

		Clinical Knowledge Score
Theoretical Knowledge Score	r	0.60
	*p*	<0.001

## Data Availability

Not applicable.

## Supplementary Materials

The following supporting information can be downloaded at: https://www.mdpi.com/article/10.3390/children9081235/s1: Table S1: Demographic and occupational characteristics independently associated with participants’ theoretical knowledge score; Table S2: Demographic and occupational characteristics independently associated with participants’ clinical knowledge score; Table S3: Demographic and occupational characteristics independently associated with participants’ total knowledge score; Figure S1: Participants’ total knowledge score.
