# Supplementary material for: Investigation of Healthcare Professionals’ Knowledge of Evidence-Based Clinical Practices for Preterm Neonatal Skin Care—A Pilot Study
Source: Children (Basel). 2022 Aug 16;9(8):1235. doi: 10.3390/children9081235 (PMC9406586; doi:10.3390/children9081235)

Table S1. Demographic and occupational characteristics independently associated with participants' theoretical knowledge score.

		β+	SE++	p
**Gender**				
	Male (reference)			
	Female	4.86	3.69	0.136
**Age**				
	20-40 (reference)			
	41-50	5.78	4.10	0.162
	51 and above	10.85	4.95	**0.041**
**Healthcare profession**				
	Midwife/Nurse (reference)			
	Obstetrician/Pediatrician/Neonatologist	11.85	4.71	**0.013**
**Educational status**				
	Technical University degree (reference)			
	Master's degree	-4.92	5.06	0.334
	Doctoral degree	1.33	4.26	0.755
**Employment status**				
	Employee in private sector (reference)			
	Employee in public sector	-0.90	4.28	0.834
	Self-employed	1.25	4.83	0.796
**Total working experience**				
	0 - 5 years (reference)			
	6 - 10 years	-8.70	4.97	0.083
	11 – 15 years	-7.95	5.03	0.117
	16 – 20 years	-6.81	5.84	0.247
	more than 20 years	-7.39	6.54	0.261
**Geographical area of current work**				
	Within the prefecture of Attica (reference)			
	Outside the prefecture of Attica	0.67	4.59	0.885
**Level of healthcare of current work**				
	Primary health care (reference)			

	Secondary health care	3.68	4.26	0.389
	Tertiary health care	-2.64	4.81	0.584
**Your theoretical knowledge about neonatal vernix caseosa, skin microbiota, bathing and clinical practices for neonatal skin care mainly derives from**				
	Undergraduate / Postgraduate studies (reference)			
	Professional experience	2.52	3.86	0.515
	Personal study and search	8.03	4.83	0.100
	Seminars/Congresses/Lectures/Courses/Other source	-2.77	4.95	0.577

+regression coefficient ++ standard error

Table S2. Demographic and occupational characteristics independently associated with participants' clinical knowledge score.

	β+	SE++	p
**Gender**			
Male (reference)			
Female	5.62	3.18	0.080
**Age**			
20-40 (reference)			
41-50	5.70	3.53	0.109
51 and above	11.12	5.12	**0.032**
**Healthcare profession**			
Midwife/Nurse (reference)			
Obstetrician/Pediatrician/Neonatologist	11.42	4.05	**0.006**
**Educational status**			
Technical University degree (reference)			
Master's degree	-6.73	4.36	0.088
Doctoral degree	1.60	3.67	0.664
**Employment status**			
Employee in private sector (reference)			
Employee in public sector	-3.35	3.68	0.365
Self-employed	0.93	4.16	0.823
**Total working experience**			
0 - 5 years (reference)			
6 - 10 years	-3.36	4.27	0.434
11 – 15 years	-8.81	4.33	0.065
16 – 20 years	-5.15	5.03	0.308
more than 20 years	-9.15	5.63	0.107
**Geographical area of current work**			
Within the prefecture of Attica (reference)			
Outside the prefecture of Attica	1.39	3.95	0.726
**Level of healthcare of current work**			
Primary health care (ref)			

Secondary health care	2.70	3.67	0.463
Tertiary health care	2.71	4.14	0.515
**Your knowledge about vernix caseosa, skin microbiota,neonatal bathing and clinical practices for neonatal skin care derive from**			
Undergraduate / Postgraduate studies (reference)			
Professional experience	5.09	3.32	0.128
Personal study and search	11.74	4.16	**0.006**
Seminars/Congresses/Lectures/Courses/Other source	-1.02	4.26	0.811

+regression coefficient ++ standard error

Table S3. Demographic and occupational characteristics independently associated with participants' total knowledge score

		β+	SE++	P
**Gender**				
	Male (reference)			
	Female	3.74	2.98	0.326
**Age**				
	20-40 (reference)			
	41-50	5.74	3.31	0.086
	51 and above	10.99	4.80	**0.024**
**Healthcare profession**				
	Midwife/Nurse (reference)			
	Obstetrician/Pediatrician/Neonatologist	11.64	3.80	**0.003**
**Educational status**				
	Technical University degree (reference)			
	Master's degree	-7.33	4.09	0.076
	Doctoral degree	1.47	3.44	0.671
**Employment status**				
	Employee in private sector (reference)			
	Employee in public sector	-2.13	3.46	0.540
	Self-employed	1.09	3.90	0.780
**Total working experience**				
	0 - 5 years (reference)			
	6 - 10 years	-6.03	4.01	0.136
	11 – 15 years	-8.38	4.06	0.062
	16 – 20 years	-5.98	4.72	0.208
	more than 20 years	-8.27	5.28	0.121
**Geographical area of current work**				
	Within the prefecture of Attica (reference)			
	Outside the prefecture of Attica	1.03	3.70	0.782
**Level of healthcare of current work**				
	Primary health care (reference)			

	Secondary health care	3.19	3.44	0.356
	Tertiary health care	0.03	3.88	0.993
**Your knowledge about neonatal vernix caseosa, skin microbiota, bathing and clinical practices for neonatal skin care mainly derives from**				
	Undergraduate / Postgraduate studies (reference)			
	Professional experience	3.80	3.11	0.225
	Personal study and search	9.88	3.90	**0.013**
	Seminars/Congresses/Lectures/Courses/Other source	-1.90	4.00	0.636

+regression coefficient ++ standard error

Figure S1. Participants' total knowledge score

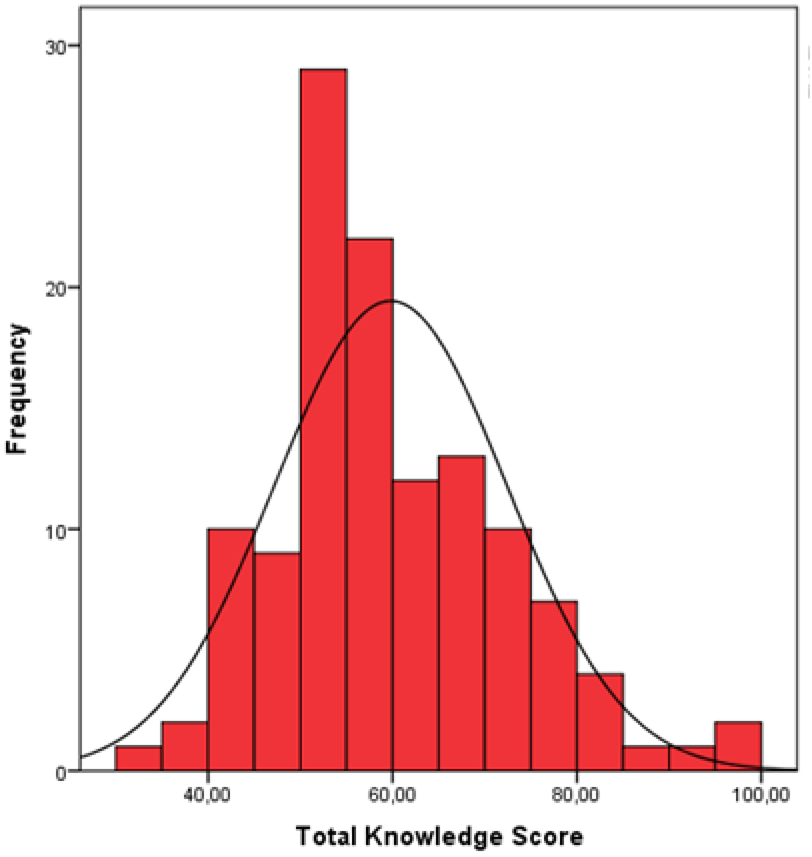

Supplement: Supplementary file 1 [file children-09-01235-s001.zip › children-1817654-supplementary.pdf]
